# Colour-Based Binary Discrimination of Scarified *Quercus robur* Acorns under Varying Illumination

**DOI:** 10.3390/s16081319

**Published:** 2016-08-18

**Authors:** Mirosław Jabłoński, Paweł Tylek, Józef Walczyk, Ryszard Tadeusiewicz, Adam Piłat

**Affiliations:** 1 Faculty of Electrical Engineering, Automatics, Computer Science and Biomedical Engineering, AGH University of Science and Technology, 30 Mickiewicza Ave., Kraków 30-059, Poland; rtad@ahg.edu.pl (R.T.); ap@agh.edu.pl (A.P.); 2 Department of Forest Work Mechanisation, Faculty of Forestry, University of Agriculture in Krakow, 46 29-listopada Ave., Kraków 31-425, Poland; rltylek@cyf-kr.edu.pl (P.T.); rlwalczy@cyf-kr.edu.pl (J.W.)

**Keywords:** pedunculate oak, scarification, viability prediction, colour image processing, machine vision, image classification

## Abstract

Efforts to predict the germination ability of acorns using their shape, length, diameter and density are reported in the literature. These methods, however, are not efficient enough. As such, a visual assessment of the viability of seeds based on the appearance of cross-sections of seeds following their scarification is used. This procedure is more robust but demands significant effort from experienced employees over a short period of time. In this article an automated method of acorn scarification and assessment has been announced. This type of automation requires the specific setup of a machine vision system and application of image processing algorithms for evaluation of sections of seeds in order to predict their viability. In the stage of the analysis of pathological changes, it is important to point out image features that enable efficient classification of seeds in respect of viability. The article shows the results of the binary separation of seeds into two fractions (healthy or spoiled) using average components of regular red-green-blue and perception-based hue-saturation-value colour space. Analysis of accuracy of discrimination was performed on sections of 400 scarified acorns acquired using two various setups: machine vision camera under uncontrolled varying illumination and commodity high-resolution camera under controlled illumination. The accuracy of automatic classification has been compared with predictions completed by experienced professionals. It has been shown that both automatic and manual methods reach an accuracy level of 84%, assuming that the images of the sections are properly normalised. The achieved recognition ratio was higher when referenced to predictions provided by professionals. Results of discrimination by means of Bayes classifier have been also presented as a reference.

## 1. Introduction

Oak (*Quercus robur* L.) is present almost all over Europe—from the Scandinavian Peninsula in the north to the Apennine Peninsulas and the Balkan in the south and from the Iberian Peninsula in the west to the foothills of the Ural Mountains in the east. In Europe, the oak is a forest-creating species, regenerated artificially; natural regeneration is confined to certain areas and only to the years of the most abundant harvest [1,2,3]. The intensive development of seedling cultures in container cultures requires the application of qualified sowing material, with an appropriate genetic potential and a very high germinating ability and yielding of equal sprouts. Obtaining certified seed material requires a number of physical actions which in turn call for knowledge of the rules governing the separation processes [4]. These are based on the recognition of physical differences in properties between various components of mixtures as well as correlations between the germination capacity and selected physical properties of seeds [5]. It should be noted that trees growing in the same stand can produce seeds that are very diverse in terms of size and weight—seeds from older trees are often smaller than from younger trees [6,7].

In the case of oak seed preparation, the multi-stage character of the processes is cumbersome. After the cleaning from heavy contamination, it is necessary to separate the seeds infested by insects and the empty ones. Then acorns are subjected to phytosanitary treatments—thermotherapy and fungicide seed treatment, aimed at reducing their mummification, which is the result of the attack of fungus [8,9]. Before sowing, in order to accelerate and align the seeding process, the acorns are subjected to scarification by cutting their ends with the cotyledons about 25% of their length on the side of the stigma. It should be noted that scarification that is too intense results in worse morphological parameters of seedlings, as in the case of damage to seedling cotyledons [10]. The results can be seen even two years after sowing [11]. Generally the relationship between the acorn size and the distribution of biomass is evident in oak seedlings [12]. Due to the low effectiveness of the mechanical methods of acorn separation with the use of classical separation features [13,14], more unconventional solutions are currently being sought [15].

We propose the use of computer image processing methods along with machine vision setup to speed up the preparation of high volumes of acorns for sowing. Image analysis implemented in machine vision systems is being used in a wide range of applications, engineering, biomedicine [16,17,18], post-harvest food processing [19] or agro-forestry [20]. Analysis of grey-level images is common, but in some cases colour information is also necessary in order to distinguish various objects of the same brightness that differ in colour. The quality of the colour information represented by hue is dependent on several factors, mainly on ambient illumination and the properties of the camera image sensor [21,22]. That is why, when dealing with colour images, stable illumination should be applied, camera setup should be calibrated during acquisition [23] or images should be normalised afterwards [24] in order to reduce the influence of changes of an uncontrolled light.

Beside video cameras, other imaging techniques are used for the prediction of properties of products. A hyper-spectral imaging technique has been proposed for analysis of moisture content uniformity [25] in maize kernels and viability prediction of castor seeds [26]. In order to examine the effects of tannin content in acorns on their fate, an infrared spectrophotometer was used in an experimental setup [27] for non-destructive measurement. A destructive method for complete classification of *Quercus ilex* and *Quercus suber* by gas chromatography-mass spectrometry was reported by [28]. 3D scanning [29] is also used to generate accurate 3D models for size and shape analysis, and the authors report that it is as accurate as digital image analysis but more time consuming than micrometer measurement. Other work [30] describes the analysis of two perpendicular views of an egg that allows for prediction of its mass by computing the area and radius. Log-polar representation and neural networks were used to perform an accurate assessment of wheat grain shape [31]. Another interesting approach for the vision-based assessment of fruit maturity is based on the development of custom colour scales in Lab colour space, which makes use of support vector machines and regression [19]. The importance of image normalisation under uncontrolled illumination has been recognised during the development of a computer-based system for crop and weed segmentation [32]. Normalised Excessive Green component was derived from input data for robust detection of plants in an outdoor environment during stages of sun activity.

The work presented here is aimed at analysing the effectiveness of acorn viability prediction using computer vision methods with colour images. In particular, an evaluation of the accuracy of binary separation of acorns using components of popular red-green-blue (RGB) and perception-based hue-saturation-value (HSV) representations [33,34] of sections of scarified acorns has been performed. The article presents a comparison of the following types of discrimination: prediction by experienced professionals and colour-based computer binary separation on sequences of colour images acquired by two types of cameras, each with different illumination conditions. The first used machine vision camera with uncontrolled spot light and was open to ambient illumination. The second used commodity camera equipped with controlled illuminator and was not exposed to external light. The contribution of the work is also the development and evaluation of an automated computer-based method of white balance and normalisation of brightness aimed at equalising images of sections. Both, non-normalized and normalised images of sections were subjected to binary discrimination and results were provided in Section 3. Predictions obtained by means of naive Bayes classifier trained using supervised learning technique were also presented for comparison.

## 2. Materials and Methods 

The setup used for collecting data in the experiment consisted of a 5 Mega-Pixel CCD machine vision camera (JAI BB-500GE, JAI Ltd., Yokohama, Japan), 12 mm fixed-focal length lens (HF12,5SA-1/1,4, Hujinon, Fujifilm Corp., Saitama, Japan) and custom directional single LED (Light Emitting Diode) spot illuminator. The illuminator did not contain a diffuser but it consisted of the lens for focusing the stream of light on the surface of an acorn aligned to the plane of the holder. The illumination of the sections captured by the camera varied due to the following reasons: (a) the setup was exposed to the ambient light in the greenhouse and varying weather conditions; and (b) the thermal effect of the LED working continuously for few hours without cooling device attached. These factors meant that the illuminance and colour temperature of the resultant stream of light should be treated as variable in this setup. Images were registered in raw format, using a full bit-depth resolution of 12 bits in order to enable further rectification and normalisation. The usable size of region of interest (ROI) circumscribed on an acorn was about 0.8 MPix. Images were finally saved in 48-bit RGB format.

Concurrently, a commodity camera (Nikon D600, Nikon Corp., Tokyo, Japan), working in manual mode, equipped with Mikro-Nikkor 105 mm zoom lens and professional circular illuminator with multiple LEDs distributed on the ring was used to capture high resolution pictures. The illuminance was fixed by the controller and colour temperature was determined by the diffusing filter covering LEDs. The setup was screened from an ambient light, so illuminance and colour temperature should be treated as constant. However, lens focus and exposure time (ranging from 1/150 to 1/13 of a second) were adjusted manually for each section separately. ISO sensitivity was fixed to the value 125. In this case, the average resolution of clipped images was much higher i.e., 6.4 Mpix. Colour images were stored in 16-bit raw format, then converted into 24-bit RGB format (i.e., 8-bits per component) and saved as high-quality JPEG files. During this stage, a histogram of each section has been normalized in order to enable visual inspection on a calibrated display which is the subject of a separate study on the morphology of mummification changes that appear in the sections.

**Figure 1:**
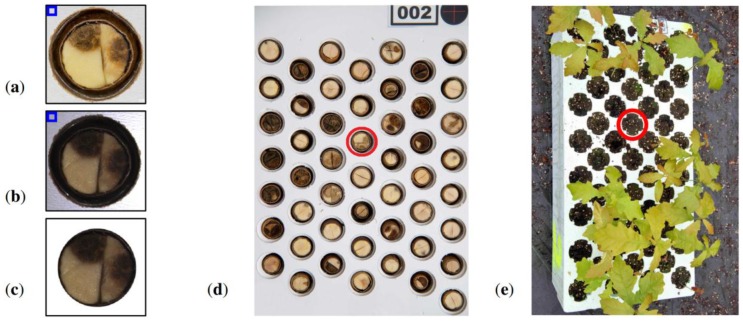
Images of scarified acorn *Id.* = 75 acquired during the experiment: (**a**) captured by high resolution camera; (**b**) captured by machine vision camera; (**c**) segmented section used in computer analysis; (**d**) cassette number 2 containing 50 scarified acorns; (**e**) germination status in container number 2. Legend: *Id.*—identifier of subsequent acorn processed during the experiment, blue rectangles—mark reference areas used for normalisation, red circles—point acorn *Id.* = 75 placed in the cassette and in the container to which it has been sown.

Sample images of both types can be seen in Figure 1a,b. Additionally 8 collections, each containing 50 sections, were captured by high resolution camera (see Figure 1d). These were handed to experts for evaluation. Germination status was also registered in a form of an image (see Figure 1e) in order to collect experimental data. Elaborated results of germination were used for computing the accuracy of subsequent predictions of viability, both manual and automatic.

Following the stage of acquisition and data formatting, subsequent stages of image post-processing were applied: (a) white balance; (b) normalization; (c) colour space transformation; (d) segmentation (determining rectangular and circular ROIs); (e) computation of scalar features; and (f) prediction of viability and analysis of results combined with experimental germination data. Seven components, hue, saturation, value, red, green, blue, and grey level (represented by following symbols: *H*, *S*, *V*, *R*, *G*, *B*, *Y*) have been used to determine scalar features for discrimination. Besides proprietary camera software, a computing environment MATLAB R2013a (The MathWorks Inc., Natick, MA, USA) was used for image processing, data analysis and visualisation of results. Adobe Photoshop Lightroom (Adobe Systems Inc., San Jose, CA, USA) software was used for producing high resolution images.

The known method of non-destructive assessment of acorn viability is visual evaluation of mummification changes of scarified acorns. For this reason it has been assumed in the research described in this paper that measuring properties of cotyledons allows for predicting viability. The rules applicable to evaluation of seeds in the State Forests follow standards introduced by the International Seed Testing Association (ISTA). Three fractions (healthy, partly spoiled, spoiled) [13,35] are defined. However, our vision-based algorithm is being designed for controlling the automaton used for scarification, which is common procedure in container nursery production. In this case, empty cells are not admissible due to economic reasons. The expected level of germination capability of partly spoiled seeds equals about 50%, which is too low for this type of production. Therefore, we claim that partly spoiled and spoiled seeds should be excluded from sowing into containers. Moreover, reference germination data, which is the result of the experiment, is binary, so partly spoiled and spoiled acorns can remain in a single category: non-germinating. After this stage, they can be subjected to further separation (spoiled, partly spoiled) if necessary. In accordance with the above principles, the overall number of scarified acorns was 400.

Accuracy *ACC* was used to assess performance of binary classification based on particular scalar features of the sections. The results were compared later with germination data and professional evaluations of 400 acorns given by 4 experts in Table 1, that includes prediction results (*TP*—True Positive, *TN*—True Negative) and overall accuracy *ACC* explained by Equation (2) in Section 3. Each expert evaluated 8 printouts of cross-sections (at resolution about 0.16 MPix per acorn) containing collections of 50 acorns as presented in Figure 1d.

In the typical section of pedunculate oak (*Quercus robur* L.) acorns, two cotyledons inscribed in a darker pericarp are visible. Usually they divide circular section into halves with a darker edge. It happens that cotyledons significantly differ in size (see Figure 1d: row 2, column 4). In such a case an edge that separates them, takes the form of an arc. Additionally, cotyledons may be cracked due to accidental crushing that may happen during preparation (see Figure 1d: row 1, column 5). Mummification changes usually appear as dark blobs within areas of particular cotyledons like in Figure 1c. When mummification level is very high, distinction of particular cotyledons is difficult or even impossible due to low brightness and homogeneous texture.

Thus, to compute the scalar features of each section of a scarified acorn, we propose to accumulate pixels values within segmented areas of sections according to Equation (1), where mask *E* of the section is binary, and *P^C^*(*x,y*) designates single components *C* of pixels (*x,y*) in a particular colour space (RGB, HSV) or grey. The level of grey was derived from components of RGB space as follows: *Y* = (0.299*R + 0.587*G + 0.114*B). All seven components were downscaled to a unit range (0.0, 1.0). In order to avoid the impact of varying sizes of acorns, the accumulated value is divided by the area *A_E_* of the section mask obtained during the segmentation stage.

(1) $$Fav^{C}=\frac{1}{A_{E}}\sum_{\left(x,y\right)}P_{\left(x,y\right)}^{C}E_{\left(x,y\right)}$$

Segmentation was performed in two stages. At first, the biggest dark object within the holder of the scarified acorn was detected using custom blob analysis software—grey level images were used at this stage. This allowed for clipping images and determining rectangular ROI for clipping as shown in Figure 1a,b. In the next step, circular mask circumscribing cotyledons and adjacent pericarp were fitted automatically by means of a circle detection procedure using popular parametric representation in Hough space: radius and coordinates of the center. Due to the presence of errors introduced by defects of pericarp caused by cutting the acorn, radii and centers of all masks were revised and some were fine-tuned manually.

**Figure 2:**
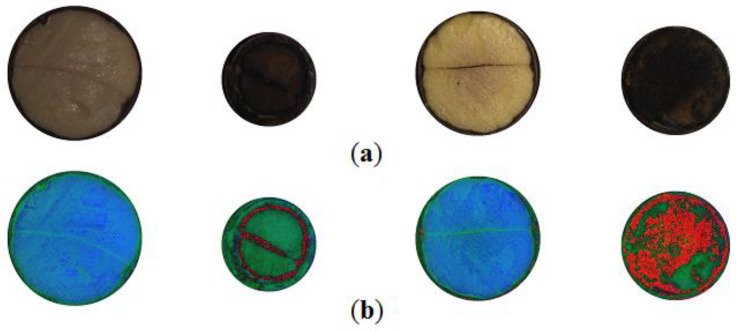
Selected segmented cross-section, *Id.* (from the left): 197, 200, 201, 206: (**a**) images captured by machine vision camera; (**b**) preview of HSV components rendered into RGB format.

During analysis of images HSV in colour space, the hue component needs additional handling, as it is represented as an angle constrained to (0.0, 1.0), scaled down from a circular angle range (0°, 360°). Because the observed hue values aggregate close to 0.0, additional pre-processing was performed by applying the offset 0.5 and modulo function: *P^H^* = mod(*H* + 0.5, 1.0). This prevents hue values from crossing the border between 0.0 and 1.0 that represent adjacent red hues. Otherwise, binary discrimination with a single threshold would not be possible because the scale of hue coordinates is not monotonic within the region where actual values of hue appear. The effect of toggling hue can be clearly seen in Figure 2b. Some regions of similar hue gain a high value whilst in other, it remains low. These two regions are rendered as intense red or bluish and greenish colours.

**Figure 3:**
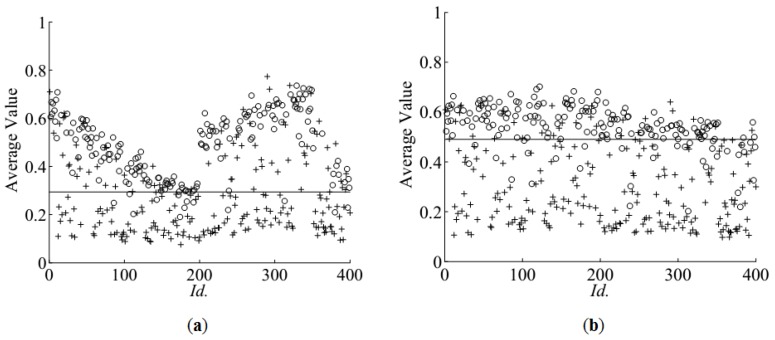
Reference germination data (experimental results) overlaid on *Fav*^V^ (average value component in HSV-space images captured by machine vision camera): (**a**) average value of non-normalized images of cross-sections; (**b**) average value of normalized images of cross-sections. Legend: ‘o’ germinating, ‘+’ non-germinating, ‘−’ level of separation threshold rendering maximum accuracy.

Figure 3a shows *Fav^V^* for images of acorns transformed into HSV space—averaged values of the value component within circular areas of sections. Germinating ones are marked with an ‘o’ symbol, and the others with a ‘+’. One should note that for each input image, the identifier *Id.* in the range (1, 400) has been assigned in order to mark subsequent acquisitions. This character is used to track changes of ambient illumination during image acquisition across a few hours of the experiment performed outside the laboratory, i.e., in a greenhouse facility where seeds were sown immediately after scarification.

**Figure 4:**
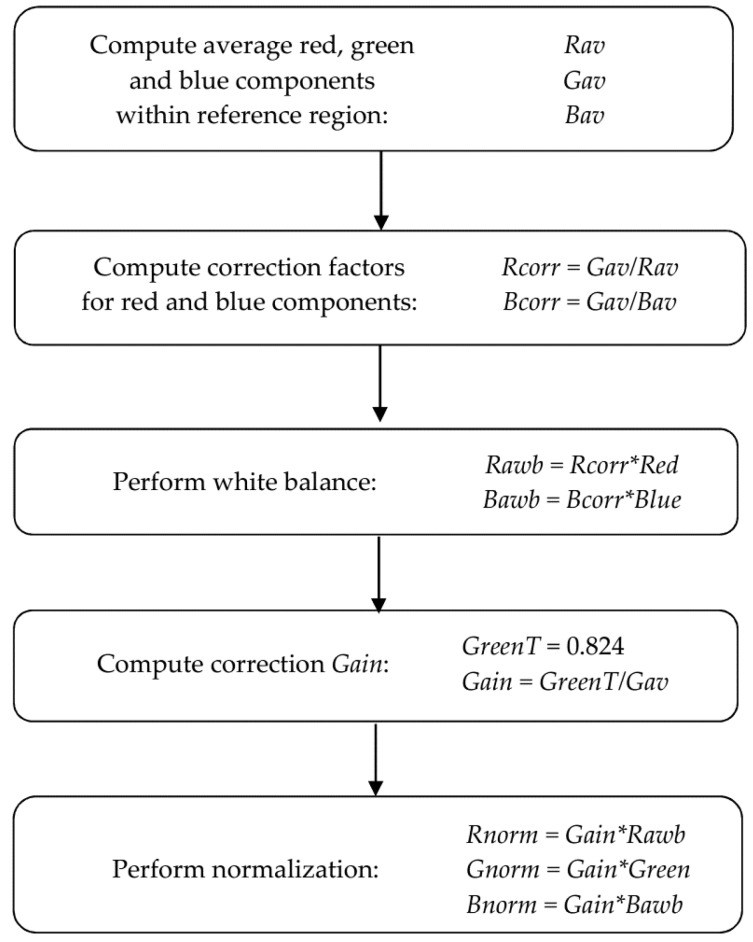
The diagram of the algorithm of an automatic white balance and brightness normalization.

It can be seen in Figure 3a that the appearances of the sections differ significantly due to varying illumination and impact of ambient conditions. In order to equalise the values of the colour components, a two-stage rectification method was used. Firstly, white balance was applied and, next, brightness normalisation was performed on RGB components by application of the *Gain* factor. The first reduces the diversity of hue information, while the latter equalises changes of overall brightness of subsequent images. Both stages, colour balance and normalisation, were executed for each input image in single pass of the algorithm presented in Figure 4.

The holder used for storing a scarified acorn at fixed distance from the camera was made of white fiberboard. Rectangle fraction of this component (100 × 100 pixels) located in left-upper corner of an image (see Figure 1a,b) containing segmented section was used as a reference marker. It allowed balancing the colours and computing of the correction *Gain* that is the ratio of desired *GreenT* = 0.824 value fixed below the upper limit and the average of green component within reference region. This prevents the saturation of bright pixels. In hyper-spectral analysis reported by [25], a white Teflon tile was used as a reference for correcting the effect of light source variations during multiple scans. In order to reduce the impact of light variability, a simplified method without a reference tile has been used in a machine vision system for seed identification [15]. The reference material we use does not allow for exact restoration of absolute colour values according to the rules of colorimetry. It enabled however, to equalise colour components so as to reduce their variability throughout the duration of the experiment.

**Figure 5:**
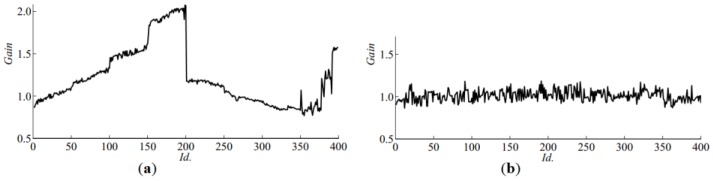
Value of correction gain factor in automatic normalization of images: (**a**) acquired by machine vision camera; (**b**) captured by high resolution camera.

**Figure 6:**
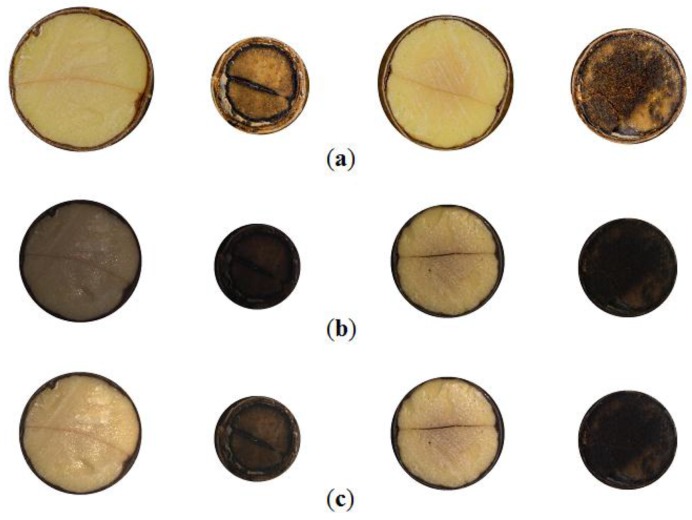
Selected segmented cross-section, *Id.* (from the left): 197, 200, 201, 206, germinating: 197, 201; non-germinating: 200, 206: (**a**) images of sections captured by high resolution camera; (**b**) images of sections captured by machine vision camera; (**c**) normalized images.

Both steps were performed automatically on a whole sets of 400 input images captured using both setups. Figure 5a shows the gain used for correcting brightness of images captured by machine vision camera. In the middle of the experiment (sample *Id.* 200), a falling slope marks the sudden increase of image brightness. The effect of the automatic normalisation can be seen in Figure 6b,c. After normalisation, according to Figure 3b, *Fav^V^* (average *V*) of the germinating acorns aggregates above the value 0.5 whilst non-germinating ones remain scattered over a wider range. Correction *Gain* for images captured by the high resolution camera can be seen in Figure 5b. On average, the brightness does not change significantly in time thanks to usage of controlled illumination and manual adjustment of exposure during acquisition. However consecutive frames differ from each other due to separate exposure times and initial normalization of histograms optimised for extended visual investigation of sections. Proposed equalisation of colours and normalisation of brightness enable to perform discrimination of features by thresholding, even though acquisition of images was not subjected to the same standards. In the first case variability was introduced by uncontrolled illumination, whilst in the latter, it was the result of manual correction of exposure and normalisation of histograms. Moreover, 41 acorns in *Id.* range (351, 391) were too big to fit into the holder. During image acquisition, they were fastened by another support made of the Styrofoam, which reflects light in a different way. This explains the presence of steep slopes at the end of the graph in Figure 5a.

## 3. Results and Discussion

In order to perform binary classification, accuracy *ACC* = max(*acc*(*Fav*^C^,*T*)) defined by Equation (2) was used as a measure of performance [17,36,37], where average components *Fav^C^* of all sections and *T* fall into range (0.0, 1.0). It takes into account both the positive and negative predictions equally. The best threshold *Thr* that divides the 400 sections into two classes is the level *T* for which the accuracy of discrimination using particular feature *Fav^C^* (where *C* designates one of seven components: hue, saturation, value, red, green, blue or grey) gains maximum value. It is searched by subsequent application of *T* level starting from 0.0 and incremented with equal step 1/255, which is correct for popular 8-bit representation.

(2) $$acc(Fav^{C},T)=\frac{TP(Fav^{C},T)+TN(Fav^{C},T)}{P+N}$$

**Figure 7:**
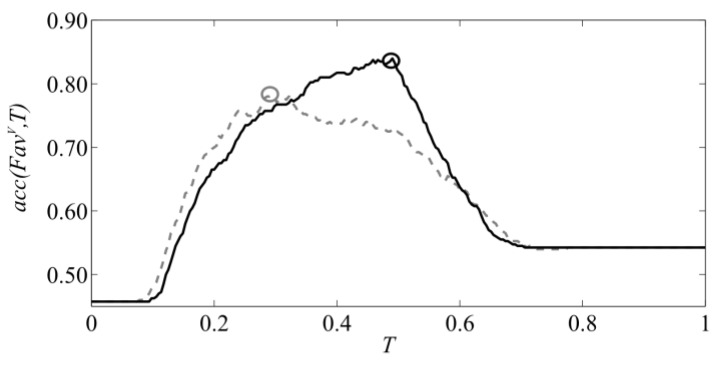
Graph of *acc*(*Fav*^V^,*T*) computed for images captured by machine vision camera: before normalization (dashed line), after automatic normalization (solid line).

Overall, the number of germinating acorns is *P* = 183, whilst the number of non-germinating is *N* = 217. By applying particular level of *T* to all 400 values of particular feature *Fav^C^*, one can obtain the number of true positive predictions of viability (*TP*) and false positive (*FP*) that sum up to the *P* value. Accordingly, for feature values that fall below the threshold, one can obtain true negative (*TN*) and false negative (*FN*) predictions. The value that produces the maximum accuracy *ACC* is considered here the threshold that separates the acorns into two classes: healthy and spoiled. In Figure 7, this point is marked with the ‘o’ character.

The best accuracy *ACC* for all 7 components is presented in Table 2: hue, saturation, value, red, green, blue and grey. For all features computed on images acquired by machine vision camera, normalised images give better accuracy. In general, the best result was achieved for value, red and grey components and slightly worse for green, blue and hue. Hues of healthy acorns gather between yellow and green.

Distribution of germinating and non-germinating seeds across the range of saturation shows that this feature is not well suited for discrimination. Analysis of features derived from images captured by high resolution camera shows that the impact of automatic normalisation is less significant. The overall performance expressed in accuracy *ACC* is also smaller than for low resolution images. The average saturations (*Fav^S^*) of germinating and non-germinating seeds are also mixed. However, for this set of images, germinating ones tend to have saturation smaller than the others. Therefore, while analysing the saturation-based feature, values were modified according to the Equation F*av^S^ = 1 − *Fav^S^*. For this reason the * symbol appears in bottom rows of Table 2 and Table 3. Maximum accuracy for high resolution images was obtained for normalised hue and green.

Overall, improvement of accuracy of binary discrimination after automatic normalisation of low resolution images was subsequently 6% for average value and 11% for average hue. The accuracy of prediction based on saturation and hue for high resolution images did not change significantly after automatic white balance. This can be explained by the fact that controlled illumination was used during acquisition and prior to automatic normalisation, custom normalisation of histograms was applied while formatting the images from raw data.

The impact of light intensity and its type is observable in the collected image sets. A directional spot light produces bright reflections when the moisture level of a scarified acorn is high, regardless of the presence of mummification changes. It should be noted that oak seeds fall into the ‘recalcitrant’ category. That is why, in order to retain their vitality, they require constant maintenance of their natural moisture—above 42% [38]. The traditional way of drying and storing acorns does not allow for obtaining homogeneous final moisture as the spoiled and half-spoiled seeds store water less well than healthy seeds [14]. The advantage of pictures captured with a high resolution camera is the uniformity of light produced by the circular illuminator. This means, however, that details distant from the camera are equally represented in the images. This applies particularly to spaces between the pericarp and cotyledons, between the cotyledons when they are not adjacent, and also between the pericarp and the holder in which the acorn remains during image acquisition. The intensity of these regions remained low when a directional spot light was used.

RGB and HSI colour images were examined [39] during identification of wheat grains infected by the *Fusarium* L. genus fungi. A high recognition rate of 98% was achieved for the two classifiers: ANN (Artificial Neural Network) and k-NN (k Nearest Neighbours). It should be noted that this recognition rate differs from the accuracy presented in Table 2 by reference data, even though both comply with Equation (2). Reference data for recognition rate computation is the result of a visual assessment of the images of the seeds by humans, whilst the accuracy *ACC* we gained was computed from germination data collected during the experiment. The rates presented in Table 3 outnumber the values of accuracy presented in Table 2 for each of the analysed image components. The saturation component renders the lowest rate. Except for the grey component, the rate of hue (for low resolution images) is also high for all 4 reference data sets provided by the experts. In the case of high resolution images, none of the red, green, blue, or grey components tends to dominate over the others, but the rate for the saturation component remains the lowest. The assumption is that humans consider both brightness and hue during an evaluation of scarified acorns. Moreover, they can derive this information from the topology of the cross-section, when the variability of the RGB and grey components for healthy and spoiled regions of the cross section is limited by uniform illumination.

The method of determining the thresholds and results of the analysis justify necessity of normalization. It has however some drawbacks when applied to continuously working scarification automaton. It is not well suited for practical implementations because images of sections of scarified acorns have to be processed beforehand in order to determine the value of the threshold *Thr*. This is why supervised learning has been also applied to collected images. Input data has been divided into learning set (240 sections) and test data set (160 sections). Mixtures of averaged components have been used for training naive Bayes classifier with normal distributions in three configurations of averaged components: red-green-blue (R-G-B), hue-saturation-value (H-S-V) and grey. This statistics-based method has been used e.g., for detection of skin in colour pictures [40] or for segmentation of plants [41] in the field. It allows complementary information to be exploited that possibly exists in mixtures of input components combined as vectors. Results presented in Table 4 show that accuracy of predictions on training set comply with values presented in Table 2 even though training set contained only 60% input samples in this case. The accuracy of recognition was slightly lower for the test data set. H-S-V representation allowed for constant accuracy in both steps when using normalised data. It has been confirmed that normalization of images improves accuracy of recognition in both: training and verification stage.

The Bayes classifier was also trained using decisions provided by experts as a reference. In this case recognition ratio varied from 89.2% to 97.5% for training set (normliased images). Results obtained from test data was starting with 83.8% for normalized high resolution images to 95.6% for low resolution normalized images in HSV space assuming Expert 2 as the reference. Considering all decisions from all experts (1–4), the best overall results were rendered for low resolution normalized HSV images, i.e., 95.0%, 95.6%, 93.1% and 94.4% subsequently. Corresponding values for high resolution images were 86.3%, 87.5%, 86.9% and 85.6%. Comparing these values to those presented in Table 3 one can make a general statement that the Bayes classifier is able to follow the decisions of experts better than the threshold-based separation method. On the other hand, some results of threshold-based discrimination presented in Table 2 outdo the best accuracy of Bayes classifier trained on experimental reference data. In the future work, extended analysis and in-field experiments are planned.

In order to reach a higher accuracy of automatic separation, more sophisticated representations of features and classifiers have to be sought. However, one should take into account that predictions based on single cross-sections are constrained due to the uncertain topography of the mummification changes that can vary along the length of an acorn. They do not allow for assessment of damage to the embryonic development and the root ovaries [35]. The results of research carried out on artificial excision of *Quercus variabilis* L. [42] show that parts of cotyledon closer to the apex are more important for acorn viability than those placed at distal ends.

## 4. Conclusions 

Colour-based discrimination is both fast and non-destructive as scarification is a regular procedure applied to acorns during processing. It requires a video camera and can be carried out in the field. White balance and normalisation improve the accuracy of computer-based discrimination when compared to the reference data sets: experimental germination data and predictions by professionals. The accuracy of acorn viability prediction using the presented computer-based method for automatic analysis is comparable to the typical performance of experienced professionals who assess high volumes of scarified acorns before sowing. A similar level of accuracy was achieved by using average value, red, green and grey components. The performance of other scalar features was slightly lower. When the average values of components are considered for discrimination, an increase of the resolution of the images of the cross-sections does not improve accuracy. Ambient conditions during image acquisition can disturb the quality of the features and thus worsen automatic prediction of viability. Nevertheless, it is possible to reduce the influence of external factors by automatic white balance and normalisation. The results were verified by means of supervised learning method which is more suitable for practical application.

## Figures and Tables

**Table 1:** The accuracy *ACC* of recognitions provided by particular experts.

	Expert 1	Expert 2	Expert 3	Expert 4
*TP*	175	172	168	177
*TN*	156	156	166	150
*ACC*	82.8%	82.0%	83.5%	81.8%

**Table 2:** The accuracy *ACC* of binary separation of cross-sections.

	Hue	Saturation	Value	Red	Green	Blue	Grey
**Low Resolution Images**							
Normalised	81.0%	71.8%	84.0%	84.0%	83.8%	83.3%	84.0%
Non-normalised	70.0%	65.0%	78.3%	78.5%	78.5%	80.3%	78.8%
**High Resolution Images**							
Normalised	80.0%	68.8% *	78.5%	78.5%	80.0%	79.3%	79.5%
Non-normalised	78.8%	68.5% *	76.3%	76.5%	79.3%	76.3%	78.8%

* Modified value of saturation used.

**Table 3:** Recognition rate referring to predictions provided by experts.

	Hue	Saturation	Value	Red	Green	Blue	Grey
**Low Resolution Normalised Images**							
Expert 1	90.8%	82.5%	85.8%	85.8%	87.0%	87.0%	90.8%
Expert 2	91.0%	82.8%	84.5%	84.5%	86.3%	86.3%	90.0%
Expert 3	91.0%	80.3%	87.5%	87.5%	89.3%	88.8%	92.5%
Expert 4	91.8%	84.0%	84.3%	84.3%	86.0%	86.0%	89.8%
**High Resolution Normalised Images**							
Expert 1	83.3%	75.0% *	85.8%	87.8%	87.3%	87.5%	87.3%
Expert 2	84.5%	74.8% *	86.0%	87.5%	87.5%	86.3%	87.5%
Expert 3	85.0%	75.3% *	87.5%	88.5%	89.5%	87.8%	89.5%
Expert 4	82.3%	74.0% *	83.8%	85.8%	85.3%	86.0%	85.3%

* Modified value of saturation used.

**Table 4:** The accuracy of separation by means of Bayes classifier.

	R-G-B	H-S-V	Grey
	**Low Resolution Normalised Images**		
Learning data	83.8%	82.5%	83.8%	
Test data	82.5%	82.5%	81.9%	
	**Low Resolution Non-normalised Images**			
Learning data	77.5%	80.4%	75.8%	
Test data	76.9%	65.0%	76.3%	
	**High Resolution Normalised Images**			
Learning data	79.2%	80.0%	79.2%	
Test data	75.6%	77.5%	76.3%	
	**High Resolution Non-normalised Images**			
Learning data	76.7%	79.6%	77.1%	
Test data	75.6%	76.3%	76.9%	
